# Comparison of surgical technique (Open vs. Laparoscopic) on pathological and long term functional outcomes following radical prostatectomy

**DOI:** 10.1186/1471-2490-14-18

**Published:** 2014-02-07

**Authors:** Ahmed Magheli, Jonas Busch, Natalia Leva, Mark Schrader, Serdar Deger, Kurt Miller, Michael Lein

**Affiliations:** 1 Department of Urology, Universitätsmedizin Berlin, Charité Campus Mitte and Benjamin Franklin, Berlin, Germany; 2 Stanford School of Medicine, Stanford, USA; 3 Department of Urology, University of Ulm, Ulm, Germany; 4 Department of Urology, Paracelsus-Hospital Ruit, Ostfildern, Germany; 5 Berliner Forschungsinstitut für Urologie, Berlin, Germany; 6 Department of Urology, University Teaching Hospital, Offenbach, Germany

**Keywords:** Prostate cancer, Erectile dysfunction, Incontinence, Radical prostatectomy, Laparoscopic prostatectomy

## Abstract

**Background:**

Few studies to date have directly compared outcomes of retropubic (RRP) and laparoscopic (LRP) radical prostatectomy. We investigated a single institution experience with RRP and LRP with respect to functional and pathological outcomes.

**Methods:**

168 patients who underwent RRP were compared to 171 patients who underwent LRP at our institution. Pathological and functional outcomes including postoperative urinary incontinence and erectile dysfunction (ED) of the two cohorts were examined.

**Results:**

Patients had bilateral, unilateral and no nerve sparing technique performed in 83.3%, 1.8% and 14.9% of cases for RRP and 23.4%, 22.8% and 53.8% of cases for LRP, respectively (p < 0.001). Overall positive surgical margin rates were 22.2% among patients who underwent RRP compared to 26.5% of patients who underwent LRP (p = 0.435). Based upon pads/day, urinary continence postoperatively was achieved in 83.2% and 82.8% for RRP and LRP, respectively (p = 0.872). Analysis on postoperative ED was limited due to lack of information on the preoperative erectile status. However, postoperatively there were no differences with respect to ED between the two cohorts (p = 0.151). Based on ICIQ-scores, surgeons with more experience had lower rates of postoperative incontinence irrespective of surgical technique (p = 0.001 and p < 0.001 for continuous and stratified data, respectively).

**Conclusions:**

RRP and LRP represent effective surgical approaches for the treatment of clinically localized prostate cancer. Pathological outcomes are excellent for both surgical techniques. Functional outcomes including postoperative urinary incontinence and ED are comparable between the cohorts. Surgeon experience is more relevant than surgical technique applied.

## Background

Patients with localized prostate cancer are candidates for surgery, radiation, or active surveillance. The gold standard for surgical treatment of prostate cancer is radical retropubic prostatectomy (RRP) with excellent success rates for postoperative functional and pathological outcomes [1,2].

Over the past decade, minimally invasive approaches for surgical management of prostate cancer have become increasingly utilized. For example, the acceptance of laparoscopy among urologists has risen from 54% in 2002 to 82% in 2009 [3]. In 2006, more than 5800 LRP had been performed by 50 different surgeons in Germany [4]. Although RARP is currently the most frequently performed procedure for radical prostatectomy (RP) in the United States, in Europe LRP still represents the preferred minimally-invasive treatment option, mainly due to reimbursement issues.

There is a limited body of literature comparing open RRP with minimally invasive approaches. We investigated the impact of surgical technique (open vs. laparoscopic) at a single institution (two hospital sites) on pathological and functional outcomes following radical prostatectomy.

## Methods

Between 2003 and 2007 more than 3,000 men underwent radical prostatectomy (RP) for clinically localized adenocarcinoma of the prostate at the Charité Department of Urology at two different campi, Campus Charité Mitte (CCM) and Campus Charité Benjamin Franklin (CBF). LRP was exclusively performed at CCM, while RRP was performed at CBF. In total four surgeons of two different levels of training (one surgeon with 200–500 cases and one surgeon with more than 500 cases for each surgical technique) were elected to be included in the analysis. All patients with neoadjuvant hormonal therapy, incomplete preoperative information including preoperative PSA, clinical stage, and biopsy Gleason score were excluded from analysis. After a random selection a total of 340 men who underwent RP between August 2003 and July 2007 were included in this study. For each LRP 500 and RRP 500 surgeon 85 and 82 patients were selected. Similarly for LRP 200 and RRP 200 surgeons 87 and 86 patients were selected. The two most experienced surgeons had already performed 500 cases prior to the selected study cohort. Correspondingly the two less experienced surgeons had only performed about 200 consecutive cases prior to their study cohort. One patient refused to participate. Therefore, 339 men formed the overall study population. All data were collected under an Internal Review Board (Charité ethical committee)-approved protocol and after obtaining written informed consent from all patients. Nerve sparing technique was performed based on patients’ preference and wish for oncological safety. Pathological evaluation was performed as previously described [5].

Data on long-term postoperative incontinence and erectile dysfunction were obtained via telephone interview on average 24 months (range 9–62 months) after surgery. Postoperative incontinence was evaluated by the number of pads used per day, per night and over 24 hours. Additionally, a validated questionnaire (Questionaire-Urinary Incontinence Short Form [ICIQ-UI SF]) was administered. The validated International Index of Erectile Function – 5 (IIEF-5) was used to evaluate postoperative erectile function.

Clinical stage, biopsy and RP Gleason score, extraprostatic extension, seminal vesicle invasion, lymph node invasion and surgical margin status were evaluated as categorical variables, while patient age and prostate size were considered continuous variables. IIEF- and ICIQ-UI scores were evaluated as continuous and categorical variables.

We used the chi-square test for categorical and Mann–Whitney-U test for continuous variables to compare the clinical and pathological characteristics of the two surgical cohorts. All statistical analyses were performed by a professional biostatistician (CD) using SPSS, version 17.0 (SPSS, Chicago, IL).

## Results

Clinical and pathological characteristics of the two study cohorts are shown in Table 1. There were no statistically significant differences between the two surgical cohorts with respect to patient age at the time of surgery, preoperative PSA level, clinical tumor stage, or biopsy Gleason score (Table 1).

**Table 1:** Preoperative and postoperative characteristics of RRP and LRP patients

	**Surgical technique**			
**Characteristics**	**RRP**	**LRP**	**Total**	p-value*
Patients (n)	168	171	339	
Age (yrs)				0.678 MW
Mean ± SD	62.6 ± 5.4	62.3 ± 5.7	62.5 ± 5.6	
Median (Range)	64 (43–73)	64 (46–74)	64 (43–76)	
PSA (ng/ml)				0.737 MW
Mean ± SD	10.1 ± 11.9	9.2 ± 6.9	9.7 ± 9.6	
Median (Range)	7.4 (0.6-99.0)	7.2 (0.6-50.6)	7.4 (0.6-99.0)	
Clinical stage (%)				0.525
cT1	94/121 (77.7)	120/167 (71.9)	214/288 (74.3)	
cT2	25/121 (20.7)	43/167 (25.7)	68/288 (23.6)	
cT3	2/121 (1.7)	4/167 (2.4)	6/288 (2.1)	
Biopsy Gleason score (%)				0.328
≤ 6	78/146 (53.4)	103/167 (61.7)	181/313 (57.8)	
7	52/146 (35.6)	50/167 (29.9)	1102/313 (32.6)	
8-9	16/146 (11.0)	14/167 (8.4)	30/313 (9.6)	
Pathology weight (g)				0.024 MW
Mean ± SD	58 ± 22	53 ± 20	55 ± 21	
Median (Range)	53 (27–150)	49 (21–173)	50 (21–173)	
Prostatectomy Gleason score (%)				0.533
≤ 6	49/161 (30.4)	43/171 (25.1)	92/332 (58.6)	
7	91/161 (56.5)	106/171 (62.0)	197/332 (59.3)	
8-9	21/161 (13.0)	22/171 (12.9)	43/332 (13.0)	
Extraprostatic extension	45/161 (28.0)	51/171 (29.8)	96/332 (28.9)	0.798
Seminal vesicle invasion	25/161 (15.5)	10/171 (5.8)	35/332 (10.5)	0.008
Lymph node invasion	14/156 (9.0)	3/64 (4.7)	17/220 (7.7)	0.422
Positive surgical margin	35/158 (22.2)	45/170 (26.5)	80/328 (24.4)	0.435
pT2 R1	13 (10.5)	21 (17.5)	34 (13.9)	0.006
Nerve sparing (%)				<0.001
None	25/168 (14.9)	92/171 (53.8)	117/339 (34.5)	
Unilaterally	3/168 (1.8)	39/171 (22.8)	42/339 (12.4)	
Bilaterally	140/168 (83.3)	40/171 (23.4)	180/339 (53.1)	
Time interval to interview (mo)				0.222
Mean ± SD	24 ± 16	24 ± 12	24 ± 14	
Median (Range)	17 (9–62)	22 (9–52)	19 (9–62)	

*Indicates test for comparison among the two age cohorts. All tests are chi-squared, unless stated otherwise.

*MW*: Mann –Whitney-U-Test.

pT2 R1 – organ confined disease with positive surgical margin.

Mean follow-up was 24 (± 16) and 24 (± 12) months for the RRP and LRP cohorts respectively. Examination of postoperative pathological variables demonstrated significant differences between the surgical cohorts for pathological prostate weight and proportion of men with seminal vesicle invasion (Table 2). RRP patients exhibited higher mean pathology weight compared to LRP patients (p = 0.024). Furthermore, RRP patients had significantly higher proportions of seminal vesicle invasion compared to LRP patients (p = 0.008). There were no statistically significant differences with respect to RP-Gleason score, extraprostatic extension, lymph node invasion or overall positive surgical margins (p = 0.435). For organ confined disease the rate of positive surgical margins was 10.5% and 17.5% for RRP and LRP respectively (p = 0.006). Patients who underwent RRP had significantly higher rates of bilateral nerve sparing prostatectomy (BNSRP) performed (83.3%) than patients you underwent LRP (23.4%) (Table 1).

**Table 2:** Comparison of postoperative urinary incontinence and erectile function following RP (erectile function based on the IIEF-5 score; patients with IIEF-score of 5 or greater only)

		**Surgical technique**		
		**RRP**	**LRP**	p-value*
Pads/day (mean ± SD)		0.6 ± 1.0	0.6 ± 0.9	0.872
Pad categories/day (%)				0.889
No incontinence (0-1 pads)		99/119 (83.2)	96/116 (82.8)	
Mild incontinence (2-3pads)		17/119 (14.3)	18/116 (15.5)	
Severe incontinence (3 pads)		3/119 (2.5)	2/116 (1.7)	
ICIQ-sum score (mean ± SD)		5.6 ± 4.6	5.3 ± 4.7	0.661
ICIQ incontinence categories (%)				0.071
None		26/126 (20.6)	37/122 (30.3)	
Mild		47/126 (37.3)	28/122 (23.0)	
Moderate		36/126 (28.6)	41/122 (33.6)	
Severe		17/126 (13.5)	16/122 (13.1)	
		RRP	LRP	p-value*
		**Unilateral and bilateral NSRP**		
IIEF-score (mean ± SD)		13.3 ± 6.6	11.4 ± 6.7	0.151
IIEF erectile dysfunction categories (%)				0.230
No ED	(22-25)	11/62 (17.7)	2/25 (8.0)	
Mild	(17-21)	7/62 (11.3)	5/25 (20.0)	
Mild-moderate	(12-16)	15/62 (24.2)	3/25 (12.0)	
Moderate	(8-11)	11/62 (17.7)	3/25 (12.0)	
Severe	(5-7)	18/62 (29.0)	12/25 (48.0)	

*Indicates test for comparison among the two age cohorts. All tests are chi-squared, unless stated otherwise.

MW: Mann –Whitney-U-Test.

Table 2 shows the postoperative data on urinary incontinence and erectile function. There were no statistically significant differences between incontinence (measured as pads used per day [p = 0.872 and p = 0.889] for continuous and categorical data, respectively) and ICIQ-UI sum score (p = 0.661 and p = 0.071 for continuous and categorical data, respectively). No data regarding baseline preoperative erectile function was obtained. Patients with IIEF-scores of 5 or less were excluded from the analysis. In men, who underwent either unilateral nerve sparing prostatectomy (UNSRP) or BNSRP, there were no differences in postoperative IIEF-5 scores (p = 0.151). The analysis utilizing categorical data from IIEF-5 (no ED, mild ED, mild-moderate ED, moderate ED, and severe ED) confirmed these findings (p = 0.230).

Subgroup analysis on patients with UNSRP was not feasible due to too few cases of patients in the RRP cohort who underwent a UNSRP and had an IIEF-score of 5 or greater (n = 1). Analysis of patients who underwent BNSRP only was not feasible due to too few cases of BNSRP in the LRP cohort.

Table 3 illustrates our data on postoperative urinary incontinence based on pads used per day and ICIQ-sum score stratified by experience of the surgeon. Although no differences were apparent with respect to postoperative incontinence based on analysis utilizing pads used per day (continuous data: p = 0.207, stratified data: p = 0.776), there were significantly higher postoperative incontinence rates among surgeons with less experience compared with surgeons with more experience, irrespective of the surgical technique applied (continuous data: p = 0.001, stratified data: p < 0.001).

**Table 3:** Comparison of postoperative urinary incontinence following RP stratified by experience of the surgeon

	**Experience of the surgeon**				
	**LRP 200**	**RRP 200**	**LRP 500**	**RRP 500**	p-value*
Pads/day (mean ± SD)	0.7 ± 0.8	0.8 ± 1.2	0.6 ± 1.0	0.5 ± 0.9	0.207
Pad categories/day (%)					0.776
No incontinence (0-1 pads)	44/53 (83.0)	45/57 (78.9)	52/63 (82.5)	54/62 (87.1)	
Mild incontinence (2-3 pads)	9/53 (17.0)	10/57 (17.5)	9/63 (14.3)	7/62 (11.3)	
Severe incontinence (3 pads)	0/53 (0)	2/57 (3.5)	2/63 (3.2)	1/62 (1.6)	
ICIQ-sum score (mean ± SD)	5.9 ± 4.6	7.2 ± 5.1	4.8 ± 4.7	3.9 ± 3.3	0.001
ICIQ incontinence categories (%)					< 0.001
None	14/55 (25.5)	8/64 (12.5)	23/67 (34.4)	18/62 (29.0)	
Mild	10/55 (18.2)	22/64 (34.4)	18/67 (26.9)	25/62 (40.3)	
Moderate	25/55 (45.5)	17/64 (26.6)	16/67 (23.9)	19/62 (30.6)	
Severe	6/55 (10.9)	17/64 (26.6)	10/67 (14.9)	0/62 (0)	

*Indicates test for comparison among the two age cohorts. All tests are chi-squared, unless stated otherwise.

*MW*, Mann –Whitney-U-Test.

## Discussion

A lengthy learning curve, ergonomics associated with instrumentation, and the requirement for expertise in laparoscopic surgery are the most important factors that make LRP a challenging technique [6-8]. These factors have influenced the widespread introduction of RARP mainly in the US. However, LRP is still the most commonly used minimally invasive treatment option for prostate cancer in Europe.

We found that surgical technique was not associated with pathological outcomes. Furthermore, we demonstrated that there was no statistically significant difference with respect to postoperative incontinence between the study cohorts. Due to imbalances in nerve sparing technique between the study cohorts, the results on postoperative erectile dysfunction do not suggest that one technique is superior to the other.

Our results regarding pathological outcomes– especially the overall positive surgical margin rate – are in concordance with the currently available literature. In a large series of LRP patients, Anastadiasis showed a positive margin rate of 28.6 and 26.5% for RRP and LRP, respectively [9]. Rassweiler et al. presented a series of 219 RRP and 219 LRP a positive surgical margin rate of 29.7 and 21.0%, respectively [10]. In a recent review comparing retropubic, laparoscopic and robot-assisted RP, Ficarra et al. reported positive surgical margin rates between 11.0 and 39.4% for LRP and between 11.0 and 40.0% for RRP [11]. The study with the highest quality evidence (level 1b) demonstrated that positive margin rates were 21.6% in the RRP and 26.0% in the LRP cohort [12]. This is the only study to date to present evidence concerning the impact of surgical technique on intraoperative and postoperative outcomes in open and laparoscopic RP. One major problem in performing a randomized trial to investigate different surgical techniques is that most patients do not wish to be randomized to a specific operating technique. Rather, they prefer to benefit from the latest surgical technique or have the surgery performed by a surgeon to whom they were specifically referred [11]. Overall, comparison of the different studies regarding pathological outcomes is challenging due to significant variation in study cohorts. Furthermore, the majority of studies presented overall rather than stage-stratified positive margin rates, which limits comparability. Nevertheless in our study the rate of positive surgical margins for patients with organ-confined disease, was higher among LRP patients (17.5% vs. 10.5%; p =0.006) than the RRP cohort. It remains unclear whether this higher rate transfers into higher rates of biochemical recurrence.

In our study, continence rate based on pads used per day was 83.2% and 82.8% for RRP and LRP respectively. Using the ICIQ-sum score to measure incontinence, we demonstrated the following rates of incontinence for RRP and LRP patients: 20.6%, 37.3%, 28.6% and 13.5% for none, mild, moderate and severe incontinence respectively in the RRP cohort and 30.3%, 23.0%, 33.6%, and 13.1% for none, mild, moderate and severe incontinence respectively in the LRP cohort. Interestingly, very few studies to date have compared functional outcomes data regarding RRP and LRP. In fact, only one study has been published using a validated questionnaire [13]. However, the aim of this study by Poulakis et al. was to evaluate functional outcomes of older patients (70 years and older). Postoperative continence is worse in older patients; therefore comparison to our study is not feasible. However, Poulakis et al. demonstrated significantly higher postoperative incontinence rates following LRP compared to RRP.

Touijer et al. demonstrated a 2-fold higher risk of patients undergoing LRP to be incontinent compared to patients undergoing RRP in their high-quality study [14]. However, in the most current meta-analysis by Ficarra et al., it was found that continence rates after RRP and LRP were similar (p = 0.56) [11]. Continence rates varied between 48%-89.0% and 75.0-92.9% for LRP and RRP, respectively. A major limitation is that the data are inconsistent with varying definitions of incontinence, mainly based on pads used per day and in many cases obtained by phone interview. Our results, however, show continence rates of 80% or higher for LRP and RRP based on pads used per day and are in concordance with the majority of published studies, which also show no statistically significant difference between postoperative incontinence rates after LRP and RRP.

Evaluating the impact of experience of the surgeon on postoperative incontinence, we found out that evaluating pads used per day did not show any significant differences on postoperative outcome. However, looking at the postoperative ICIQ-sum score, surgeons with more experience (> 500 cases) produced significant better results than surgeons with less experience (< 500 cases). These results are especially interesting, because they stress the importance of sufficient evaluation of postoperative outcome based on validated questionnaires. Our findings are in concordance with the findings of other investigators who have demonstrated that experience of the surgeon is one of the most important factors impacting postoperative functional and ontological outcome irrespective of the surgical technique applied [15,16].

Another interesting additional aspect of our analysis is that – despite higher rates of bilateral nerve sparing technique in the RRP compared to the LRP group – urinary continence rates postoperatively did not differ between the cohorts. According to previous studies, continence rates were higher in patients who underwent a nerve sparing procedure [17]. One could argue that continence rates would have been potentially higher in the LRP cohort if more bilateral nerve sparing procedures had been performed. However, due to the limited number of patients, subgroup analyses could not be performed and this issue remains speculative.

Data obtained on postoperative erectile function is even more challenging to compare due to significant differences in study design and preoperative baseline erectile function of the different cohorts. Given the fact that preoperative data on erectile function was not obtained in our study, comparison to other studies is impossible. However, since postoperative data on erectile function was collected in the current analysis, we considered it important to present this data. In our cohort, there were no differences in postoperative erectile dysfunction between the two cohorts irrespective of nerve sparing procedure performed (UNSRP and BNSRP vs. BNSRP only). Subgroup analysis for patients with UNSRP could not be performed because of the limited number of patients with UNSRP in the RRP cohort. However, despite limitations in comparability of the study cohorts, erectile functional outcome seems similar for RRP and LRP patients.

There are limitations to the current study. Firstly, we did not investigate biochemical outcomes and perioperative complication rates associated with the two surgical techniques. Secondly, preoperative data on urinary incontinence and erectile function was not available. While this may not play an important role for urinary incontinence it could have impacted the data on postoperative erectile function significantly. Taking into account that approximately 30-40% of the male population in the same age range like our study cohort is considered to suffer from erectile dysfunction, it would have been of significant clinical importance to have the postoperative erectile dysfunction data compared to the erectile status preoperatively. Thirdly, a selection bias may be present for the type of surgical technique offered to each patient as well as different patient referral patterns that may exist for each surgeon. Only a randomized study incorporating the two surgical approaches for RP would be likely to produce better comparable results. Furthermore, compared to other contemporary series, the number of cases included in our study is relatively low and cases with BNSRP are less frequent than in other series published in the literature. Another limitation is that pathology reports were provided by different uropathologist from two campi. Although theses pathologist work at the same institution there might be differences in evaluation of the specimens.

Overall, our study is in concordance with the current literature proving that clinical outcome following RP is independent of the surgical technique applied. Furthermore, independent evaluation of postoperative outcome by blinded investigators seems necessary.

## Conclusions

RRP and LRP have been proved to produce excellent pathological and functional outcomes. In our cohorts, the overall positive SM rate did not differ significantly for RRP and LRP patients. Furthermore, we did not identify any statistically significant differences with respect to functional outcome including postoperative incontinence and erectile dysfunction. However, more experienced surgeons had higher postoperative continence rates bases on ICIQ-scores irrespective of the surgical technique applied. Further prospective studies including additionally patients who undergo RARP with more extended follow-up and data on disease recurrence are warranted to determine differences in clinical outcome and potentially identify the most advantageous surgical technique for RP.

## Abbreviations

BNSRP: Bilateral unilateral nerve sparing prostatectomy; ED: Erectile dysfunction; ICIQ-UI SF: Questionaire-urinary incontinence short form; IIEF: International index of erectile function; LRP: Laparoscopic radical prostatectomy; PSA: Prostate-specific antigen; RP: Radical prostatectomy; RARP: Robot-assisted radical prostatectomy; RRP: Retropubic radical prostatectomy; UNSRP: Unilateral nerve sparing prostatectomy (BNSRP).

## Competing interest

The authors declare that they have no competing interests.

## Authors’ contribution

Conception and design: AM, SD, ML, acquisition of data: JB, AM, ML, analysis and interpretation of data: JB, AM, SD, ML, drafting of the manuscript: JB, AM, SD, ML, NL, critical revision of the manuscript for important intellectual content: JB, AM, MS, SD, ML, KM, NL, statistical analysis: JB, AM, CD, obtaining funding: no funding, administrative technical or material support: KM, MS, supervision: ML, SD, KM, MS. All authors read and approved the final manuscript.

## Pre-publication history

The pre-publication history for this paper can be accessed here:

http://www.biomedcentral.com/1471-2490/14/18/prepub
